# Community Options to Fund Aging Services: A National Study to Track Local Initiatives

**DOI:** 10.1093/geroni/igab046.1927

**Published:** 2021-12-17

**Authors:** Athena Koumoutzis, Jennifer Heston-Mullins, Pamela Mayberry, Robert Applebaum

**Affiliations:** Miami University, Oxford, Ohio, United States

## Abstract

The majority of federal support for older people needing in-home services and supports comes from the Medicaid program. However, less than 10% of older people are eligible for Medicaid and to receive long-term services, a person must have a severe disability. Many older people with moderate levels of disability or those who are not impoverished are not eligible. In response to these system limitations, some counties across the nation have developed alternative funding strategies, such as property tax levies, to better serve older members of their communities. After identifying 15 states with such initiatives, a survey was distributed to 414 contacts within these states, with a response rate of 55%. Respondents included organizations such as area agencies on aging, councils on aging, and county departments on aging. Local funding varied within and across states, with annual funding ranging from $8,000-$47 million. Most commonly provided services with local funds include home-delivered (81%) and congregate (73%) meals, transportation (61%), and homemaker services (49%). A majority of programs (63%) indicated that local funds are used to provide at least one family or friend caregiver service. This study is the first compilation and description of locally-funded elder service initiatives in the U.S. Locally-funded initiatives can help older people with long-term services needs continue to live in their own homes and communities. On the other hand, some have raised questions about whether this is a good approach to funding aging services, raising concerns that this will lead to further inequities across states and communities.

